# Pro-environment behavior in China: unveiling the role of self and collective efficacy, individual and social norms

**DOI:** 10.3389/fpsyg.2025.1696917

**Published:** 2025-12-17

**Authors:** Lizhen Qiu

**Affiliations:** Hainan Vocational College of Politics and Law, Haikou, Hainan, China

**Keywords:** pro-environmental behavior, self-efficacy, collective efficacy, personal norms, social norms

## Abstract

**Purpose:**

This study examined the determinants of pro-environmental behavior (PEB) in China, drawing on Social Cognitive Theory and Norm Activation Theory to explore how psychological and social factors—namely self-efficacy, collective efficacy, individual norms, and social norms—influence environmentally responsible actions.

**Methodology:**

Based on social cognitive theory, a self-administered online survey was conducted with 476 Chinese individuals. The target population consisted of residents of China aged 18 years and above, including both urban and rural participants. A convenience sampling technique was utilized and Structural Equation Modeling (SEM) was applied to examine the relation among self-efficacy, collective efficacy, individual norms, social norms, and pro-environmental behavior.

**Findings:**

Results indicate that self-efficacy, collective efficacy, individual norms, and social norms significantly and positively influence PEB. Moreover, self-efficacy mediates the relationship of personal norms, collective efficacy and PEB. Enhancing collective efficacy and aligning it with supportive social norms emerge as effective strategies to foster environmental sustainability behaviors.

**Implications:**

The findings suggest that policies and programs aiming to enhance collective efficacy and strengthen pro-environmental social norms can effectively promote sustainable behaviors in China. Educational campaigns and community-based initiatives emphasizing collective responsibility may significantly improve environmental outcomes.

**Originality/value:**

This study advances understanding of collective efficacy’s role in shaping PEB within the Chinese context, offering empirical insights for policy development and community-level interventions.

## 1 Introduction

The environmental degradation has become one of the most urgent global issues of the twenty-first century that threatening ecological balance, population health, and global economic sustainability. Intergovernmental panel on climate change (IPCC) reports that since 1990, the global emission of greenhouse gases has grown more than 50% and the average temperature has risen by 1.1 °C above the pre-industrial levels (Global Carbon Project, 2023). These effects are too extensive: increased heatwaves, floods, the loss of biodiversity, food and water insecurity. The rate of deforestation across the world is also at an alarming rate of about 7 million hectares annually with more than 1 million species becoming extinct in the next few decades (Statista, 2024; World Bank, 2022). The reefs have been destroyed by coral diseases accelerated by ocean warming, 84% of reefs worldwide have been destroyed during 2023–2025 [Ministry of Ecology and Environment of China, 2023; World Health Organization (WHO), 2021]. All these emphasize a desperate necessity of extensive implementation of pro-environmental behavior (PEB) to lessen the harmful effects on the environment and enhance sustainability in accordance with the Sustainable Development Goals (SDGs).

China is a key participant in the world environmental equation because of its industrialization level, population, and economic power. In 2023, China produced an estimated 12.6 billion metric tons of CO_2_-equivalent (around 35% of the overall global emissions), which is more than many developed economies could produce collectively [World Health Organization (WHO), 2021; United Nations Environment Programme (UNEP), 2022]. The economic and health impacts of the degrading environment in china are devastating with an estimated cost of USD 267 billion each year, while USD 112 billion is spent on health related expenses [United Nations Environment Programme (UNEP), 2022]. In addition, more than half a billion individuals in China are exposed to unhealthy air quality [Ministry of Ecology and Environment of China, 2023; World Wildlife Fund (WWF), 2022]. The World Health Organization estimates that about 4.2 million pre-mature deaths happen annually as a result of air pollution [World Health Organization (WHO), 2021; Reuters, 2024]. In addition to air pollution, water, soil pollution is also widespread: more than 80% of the rivers and 90% of the ground water sources are contaminated, and almost 60% of agricultural soil has contamination levels exceeding the limit [World Wildlife Fund (WWF), 2022; National Oceanic and Atmospheric Administration (NOAA), 2024]. Accordingly, China is the biggest emitter in the world and one of the central actors of global supply chains, so its impact on the environment has wide-ranging consequences on the global climate. These environmental crises cannot be solved through technological and policy steps alone; it would take behavioral changes. Pro-environmental behavior (PEB) is a behavior that helps to limit environmental degradation and leads to sustainability, including lowering energy usage, recycling, and sponsoring ecological efforts [Food and Agriculture Organization of the United Nations (FAO), 2020]. Researchers highlight that sustainable lifestyles by individuals and communities as well as institutional reforms are the only ways in which global environmental objectives can be attained [Food and Agriculture Organization of the United Nations (FAO), 2020; Associated Press, 2024]. This is why it is important to know the psychological, social and cultural determinants of PEB in order to develop powerful behavioral interventions.

Addressing these environmental crises requires more than technological and policy interventions—it necessitates behavioral transformation at both individual and collective levels. Pro-environmental behavior (PEB) refers to actions that minimize environmental harm and contribute to sustainability, such as reducing energy consumption, recycling, and supporting eco-friendly initiatives (Reuters, 2024). Scholars emphasize that global environmental goals can only be achieved if individuals and communities adopt sustainable lifestyles alongside institutional reforms [National Oceanic and Atmospheric Administration (NOAA), 2024; Food and Agriculture Organization of the United Nations (FAO), 2020]. Therefore, understanding the psychological, social, and cultural determinants of PEB is crucial for designing effective behavioral interventions.

While single individual efforts are crucial, collective action are also necessary to bring substantial reforms. Several ways to implement pro-environmental behavior (PEB) of an individual have been suggested and tested through earlier research. Much of the psychological research is of view that individual behavior needs modification to combat climatic changes (Nasir et al., 2023; Akhtar et al., 2022). Furthermore, the behavior of household has tremendous potential to reduce climatic changes (Bandura, 1983). Studies Bearden and Etzel (1982), and Boon-Falleur et al. (2022) elaborated the importance of individual actions in order to reduce greenhouse gas emissions. Thus, households adopting PEB, substantially lower greenhouse gas emissions (Qin et al., 2022). It is, therefore necessary to look into the factors affecting the attitude of people toward the climatic changes and their pro-environmental behavior (Cai et al., 2024; Chang et al., 2022).

China presents a particularly interesting case for studying PEB because of its collectivist cultural orientation, which prioritizes group harmony, interdependence, and shared responsibility over individual autonomy (Associated Press, 2024). Collectivist societies strongly dictate behaviors based on social expectations, group norms and collective efficacy as collective action can create significant change. This is contrary to the individualistic culture where individual efficacy and self-motivation are more eminent. Social influence and cooperation can be enhanced in China through the collectivist ethos but individual initiative can be limited when there is no collective agreement (Fritsche et al., 2018). Collectivist ethos in China enhances the possibility of collective efficacy, the belief that there is a collective effort that can lead to the improvement of the environment. It helps to encourage collaboration, supports social norms that compensate the environmentally friendly behavior, and encourages the population to engage in the common environmental effort (Boon-Falleur et al., 2022). Nevertheless, it may also limit individual agency where the collective agreement or institutional backing is low. Unlike the individualistic settings of the West, where self-efficacy and personal values prevail in the construction of behavior, this collectivist system in China suggests that social integration, moral alignment, and social responsibility are the key factors to explain why people behave and do not behave in a pro-environmental manner (Nasir et al., 2023; Akhtar et al., 2022).

The influencing factors of PEB may be individual, social and contextual (Fritsche et al., 2018). The environmental knowledge, personal values, moral norms, and psychological constructions such as self-efficacy (Steg and Vlek, 2009; Gifford and Nilsson, 2014) are individual factors whereas the social norms, peer behaviors, and cultural expectations (Cialdini et al., 1990; Bamberg and Möser, 2007) are social factors. Contextual or structural factors comprise availability of infrastructure, economic incentives, regulation policies and the availability of green technology (Gardner and Stern, 2008; Stern, 2000). This study contended that individual norms, social norms, collective efficacy, and self-efficacy are determinants that can be used to affect the PEB. Social cognitive theory views the collective efficacy as a group belief among the individual members of the group that the group will prove to be effective. Collective efficacy is the perception of capacity of combined actions to conserve the environment, in terms of PEB. It increases the motivation within a group, fosters collaboration to identify the ways to address environmental concerns, and a feeling of collective responsibility towards environmental protection (Gardner and Stern, 2008). When group members have belief in their collective ability, they are able to convince each other to participate in the pro-environmental behaviors, which facilitates the coordination and cooperation among the group members, and thus effective environmental behavior is realized (Doherty and Clayton, 2011).

Another important element is social norms, which influence the behavior through the formation of subjective attitudes and perceptions concerning what is socially acceptable and preferably desirable (Cheng et al., 2022). Social norms have much importance for pro-environmental behavior because norms define group membership and identity, encouraging individuals to adopt PEB to maintain a positive self-image. Moreover, norms exert pressure to conform, encouraging individuals to adopt PEB to avoid social disapproval. Norms can support policy initiatives, encouraging individuals to adopt PEB aligned with policy goals (Cialdini and Jacobson, 2021).

In addition to psychological and social factors, the direct experiences of climate change may affect pro-environmental behavior: people can experience extreme weather conditions, air pollution, or lack of resources. The existing literature indicates that individual experience supports cognitive and normative motivations towards sustainable action (van der Linden, 2015). In the concept of Social Cognitive Theory, these experiences can boost self-reflection and perceived efficacy, whereas in the concept of Norm Activation Theory it can trigger some kind of personal moral responsibility. Thus, experience of climate change is introduced in this study as an additional construct to investigate whether the experience of environmental degradation enhances the psychological and social mechanisms like efficacy beliefs and norms, which produce pro-environmental behavior among the Chinese.

The existing research on pro-environmental behavior (PEB) has primarily concentrated on either the psychological processes at the individual-level or the social-level determinants, and conducted their research as a detached entity instead of an integrated mechanism. Studies that apply the Social Cognitive Theory (SCT) have prioritized agency at the individual level, including self-efficacy and perceived behavioral control, as major predictors of environmental behavior (Cai et al., 2025a). On the other hand, research with Norm Activation Theory (NAT) has emphasized the ethical and social aspects of behavior and, specifically, the influence of personal and social norms on stimulating pro-environmental decisions (Cai et al., 2025b). Most of the previous researches have focused these frameworks separately, either focusing on individual psychological forces or social forces separately. Although a limited number of studies have integrated these frameworks to comprehend the influence of efficacy beliefs and social norms in developing sustainable behaviors. In collectivist cultures like China, social relations, group cohesion and collective accountability of group performance are part of the individual behavior. Therefore, the combination of SCT and NAT offers a more detailed idea about the interaction of self-efficacy and collective efficacy with individual and social norms to determine PEB. Such integration will bridge a major knowledge gap by providing a model that includes both individual motivation as well as the social background within environmental behaviors (Cai et al., 2025c). Furthermore, the mediating effect of self-efficacy is also discussed. The main aim is to examine the psychological and social predictors of PEB among Chinese people with particular emphasis given to the significance of the self-efficacy, collective efficacy, personal norms, and social norms. By combining both Social Cognitive Theory and Norm Activation Theory, this study seeks to come up with a complete concept of the combined effects of personal and social factors towards achieving sustainable behavioral effects. Based on these goals, the following research questions are addressed in this research:

1. How does self-efficacy influence pro-environmental behavior among Chinese individuals?
2. What role does collective efficacy play in fostering pro-environmental behavior?
3. How do perceived social norms affect pro-environmental behavior?
4. How do personal norms shape pro-environmental behavior in China?
5. How self-efficacy mediates the relationship among personal norms, collective efficacy and pro-environment behavior?

The use of robust empirical methods like structural equation modeling, and data collection based on a large sample of Chinese people, allow this study to present new information on the interaction between efficacy and norms to form sustainable behavior. The results not only provide theoretical frameworks but also practical policy implications to policymakers who would like to promote environmental responsibility both in individuals and the community. Therefore, the study makes a significant addition to the emerging academic debate in the field of environmental behavior, as it puts the focus on sustainable action and supporting the role of collective efficacy and social norms in the process.

## 2 Theoretical foundation

A sound theoretical framework is necessary in understanding the determinants of PEB which involves both the individual and social dimensions of behavior. The present paper is based on two well established psychological theories; Social Cognitive Theory (SCT) and Norm Activation Theory (NAT) to introduce the impact of personal norms, social norms, self and collective efficacy on pro-environmental behaviors. The Social Cognitive Theory (Bandura, 1986) is based on the argument that, behavior is influenced by the interplay of cognitive, behavioral, and environmental factors. One of the main principles of SCT is that human beings are not considered as passive receivers of environmental stimuli; instead, they are active creators of behavior that is shaped by their internalized beliefs and external feedback. Self-efficacy and collective efficacy are two constructs, which are at the heart of SCT.

Self-efficacy is how an individual believes in his ability to engage in an activity that results in the desired outcomes. Regarding environmental behavior, highly self-efficacious people take sustainable behaviors such as recycling, energy conservation or consumption reduction since they also think that their contribution to it can have a significant effect (Bandura, 1983). Collective efficacy is a concept that stretches to the group level. It means the feeling of having the capability to achieve environmental objectives that is held by a community or a group. Collective efficacy supports cooperation, mutual reinforcement and collective action, which are important in tackling systemic environmental problems (Bandura, 2000). Collectivism (such as in China) is the culture in which the effects of collective efficacy are relevant since people typically value the group cohesiveness and collective interests. SCT particularly applies to PEB since it focuses on agency, individual and collective dimensions in stimulating behavior change. It offers a structure of thinking about how the intellectual convictions of individuals on their potentials and the potentials of the community influence the willingness to act in an environmental responsible manner.

The Norm Activation Theory (Bandura, 2000; Schwartz, 1977) is the explanation on the effect of moral norms that are internalized in prosocial and PEB. NAT argues that the most immediate predictors of behavior are personal norms which are feelings of moral necessity to act in a particular manner. These individual norms become activated when people know about the aftermath of a problem and are conscious to solve this problem. When people are aware of the effect of high energy consumption of climate change (perception of causation) and they assume that they themselves ought to lower their consumption (imputation of responsibility) then their duty to act sustainably is activated. This sense of obligation becomes a guide to behavior even without the help of external incentives. Besides personal norms, NAT acknowledges the role of social norms; the expectations of the society on what is acceptable in behavior (Cai et al., 2024). The social norms can be strong motivators, especially when people are trying to achieve social approval, or they are trying to evade disapproval. NAT supplements SCT to take into consideration moral and normative aspects of behavior. Whereas SCT focuses on the beliefs about the ability to act, NAT focuses on the moral motivation and social expectations that make people to act in environmentally responsible manner. This paper develops a hybrid framework that incorporates the agency based approach with the normative pathways to PEB by applying SCT and NAT. The belief that the individual and the society possesses the capabilities to bring change through self-efficacy and collective efficacy (SCT). In the meantime, personal and social norms are the internalized moral imperatives and social pressure which govern behavior.

While both SCT and NAT have initially been created in Western, individualistic context, their implementation in the collectivist culture of China requires a cultural adjustment. The behavior of people in the society is mostly influenced by the group interests, task division and the importance of social harmony in the society as seen in collectivist societies like China (Cai et al., 2025a). In this background, the constructs of efficacy and norms take culturally oriented meanings. As an example, collective efficacy will be a more effective predictor of pro-environmental behavior than individual self-efficacy, with individuals who have a sense of their personal agency framed through the prism of group competence and collective responsibility. Likewise, the social and personal norms are closely interwoven, and moral commitments tend to mirror the demands of society and its values instead of being individual beliefs. Thus, the combination of SCT and NAT in the present study not only provides both the interaction of social and efficacy determinants of behavior but also indicates the process of reinforcing those mechanisms by Chinese collectivist orientation (Gifford and Nilsson, 2014). This conceptual translation highlights the cross-cultural applicability of the model and offers the explanatory capabilities of SCT and NAT beyond its cultural contexts of origin.

The current research goes beyond a mechanical integration of the Social Cognitive Theory and Norm Activation Theory because it directly links the central propositions of both theories in a single framework of pro-environmental behavior. SCT focuses on the bidirectional relationship between the personal cognition, behavior and the environment with cognitive determinants, especially self-efficacy and collective efficacy influencing the behavioral performance (Bandura, 1986). In comparison, NAT assumes that pro-social and altruistic acts are a result of moral obligation, which occurs when people are aware of the environmental impacts and consider that they have a personal responsibility toward them. The proposed framework conceptualized the idea that personal and social norms (as in NAT) define the moral and social impetuses, whereas self- and collective efficacy (in SCT) turn these impetuses into actual behavior by giving one’s abilities (individual agency) and the group capacity (collective agency) to reach the environmental objectives. This theoretical synthesis thereby points out how moral cognition and behavioral control jointly operate in the development of PEB, and presents a more comprehensive and dynamic model in line with the propositions of SCT and NAT.

## 3 Literature review and hypotheses

### 3.1 Pro-environmental behavior (dependent variable)

PEB is defined as an activity that is undertaken at the group or individual level with a view to preserving, conserving, or even improving the natural environment and its resources. It has been described as “environmentally friendly behaviors,” designed to prevent wastage of resource, save water and energy, employ healthy means of transportation while preserving the wildlife and environment (Cheng et al., 2022). PEB encompasses the behaviors ranging from recycling and utilization of reusable products to more complex such as lobbying for environmental policies and taking part in environmental support activities (Schwartz, 1977). PEB is driven by the fear for the environment, minimization of ecological footprint, and significance for sustainable life of the present and future generations (Clayton and Karazsia, 2020).

### 3.2 Collective efficacy (independent variable)

PEB is affected by numerous factors and collective efficacy is one of them. Collective efficacy is based on “social cognitive theory” and has significant relevance in influencing the behavior of individuals. The theory assumes that collective efficacy can enhance motivation, persistence, and resilience among group members leading to collective action (Cai et al., 2025c). When people feel that their community is capable of dealing with environmental issues, they are likely to act in a PEB” (Albright and Crow, 2019). Collective efficacy is often dependent upon the common goals and values of a group or community. A group’s commitment to environmental preservation, collective efficacy enhances actual participation in PEB to achieve the collective goals (Fatima and Azhar, 2021). When a person perceives that its group or community values and supports PEB, there is an increased likelihood to adopt PEB. Groups with high scores in collective efficacy are likely to coordinate, share resources, and work together with others to confront environmental challenges successfully (Maran and Begotti, 2021). Through normative processes, collective efficacy may also influence PEB. Groups that have high collective efficacy will be more involved in collective action to achieve environmental goals, makes it possible to accomplish tasks that are beyond the reach of an individual member (Mar et al., 2023).

There is strong empirical support regarding positive relationship between collective efficacy and behaviors towards environments (Fritsche et al., 2018; Hornung, 2022; Howe et al., 2019). Recent research highlighted the role of collective efficacy in driving sustainable actions. The study (Chang et al., 2022) found that communities with higher collective efficacy showed stronger commitment to environmental protection practices, emphasizing the power of shared beliefs in achieving collective environmental goals. The research (Qin et al., 2022) also showed that when collective efficacy is promoted then participation in energy-saving and waste-reduction actions was increased by urban residents. These results support the hypothesis that collective efficacy strengthens the motivation of a group, cooperation, and resource mobilization for the environment. It is found that communities with greater collective efficacy perform better to boost environmental initiatives.

*H1*: Collective efficacy has positive relation with pro-environmental behavior in China.

### 3.3 Self-efficacy (independent variable)

Individual or self-efficacy, “is the belief in one’s own ability to successfully complete tasks and reach goals” (Schwartz, 1977). It is based on the idea that individual who believes that it can accomplish tasks puts forth efforts and persevere in the face of challenges. Individual efficacy is a key component of motivation and plays an important role in determining behavior. Self-efficacy influences behavioral intentions, which in turn impact actual behavior. Individuals with high self-efficacy have strong intentions to engage in PEB and follow through with these intentions. It influences outcome expectations, or the beliefs about the outcomes of behaviors (Corral-Verdugo et al., 2016). Social cognitive theory is of view that individuals with high self-efficacy believe they can successfully perform tasks, including pro-environmental behaviors, which increases their motivation and perseverance in engaging in these behaviors (Lu and Wang, 2022). Individuals with high self-efficacy adopt behaviors that protect the environment as they believe they can effectively perform the necessary actions (Mackay and Schmitt, 2019).

Numerous studies and interventions have demonstrated a positive relationship between self-efficacy and PEB, showing that individuals with higher self-efficacy adopt and sustain these behaviors. The findings of the studies (Fritsche et al., 2018; Crandon et al., 2022) depict that self-efficacy is a significant predictor of health-related behaviors, which can be extended to environmental behaviors. The belief in one’s capability to affect positive change influences the likelihood of taking action. Numerous studies and reviews, such as Gifford and Nilsson (2014), Schwarzer and Luszczynska (2007), and Homburg and Stolberg (2006) provide “robust empirical support for the positive relationship between self-efficacy and PEB.” By synthesizing insights from earlier literature and theoretical foundations, it is hypothesized as;

*H2*: Self-efficacy has positive relation with pro-environmental behavior in China.

### 3.4 Social norms (independent variable)

Social norms are “the unwritten rules that guide behavior within a particular social group or community. These norms dictate what is considered acceptable or appropriate behavior in different situations” (Fatima and Azhar, 2021), hence able to influence individual attitudes, beliefs, and activities. Social norms can be either implicit or explicit and differ across cultures, societies, and social groups. In this way, they exert strong behavioral influence as one tends to act according to the social norms so that one may belong to that group, seek social approval (Maran and Begotti, 2021). Social norms theory suggests that individuals’ behaviors are influenced by their perceptions. If individuals perceive that pro-environmental behavior is the norm in their community, they engage in such behavior to conform to these social expectations (Bandura, 1986). Norm activation theory explains that social norms can activate personal norms, leading to PEB when individuals are aware of the consequences of their actions and feel a moral obligation to act.

The research conducted by Cialdini et al. (1990) on littering behavior found that social norms affected the likelihood of littering, bringing out the power of social norm in developing the behaviors. De Groot and Steg (2009) found perceived social norms to be a significant predictor of energy-saving behaviors, indicating that if individuals perceive that in their social setting, others also have practiced the said behavior, they try to save more energy. Bamberg and Möser (2007) also made a comprehensive review of factors of PEB and stated that social norms affect the behavior of people. In particular, the influence of social norms was significant in shaping the behaviors, for example, recycling and use of public transportation (Chen and Lu, 2024). As Nolan et al. (2008) have found, people were more influenced by the social norm than other informational messages. If people believed their neighbors were saving energy, then they too conserved more. Grounding the hypothesis in theoretical and empirical literature provides a strong basis for further exploration and validation of the empirical material.

*H3*: Social norms has positive relation with pro-environmental behavior in China.

### 3.5 Personal norms (independent variable)

Individual norms, often referred to as personal norms, are internalized standards of behavior that guide an individual’s actions based on their personal values, beliefs, and moral principles. These norms are self-imposed and reflect what an individual perceives as right or wrong, desirable or undesirable. They differ from social norms, which are expectations and rules within a society or group about how members should behave (Schwartz, 1977). These norms are often rooted in moral and ethical beliefs. Personal norms are determined by the understanding and beliefs to be held about the world: environmental knowledge and knowledge bearing social justice (Cialdini and Jacobson, 2021). The theory of norm activation suggests that personal norms are internalized standards of behavior emanating from one’s self-concept and moral beliefs. Such norms are activated whenever a person is aware of consequences of actions and feels a moral obligation.

Schwartz’s (1977) foundational work demonstrated that persons with strong personal norms are engaged in pro-social behaviors. Thøgersen (1996) explained that strong personal norms about recycling were closely linked to higher rates of recycling. People who had a moral sense of duty to recycle were more regular in their recycling. Harland et al. (1999) demonstrated that interventions focused on reinforcing individual norms (e.g., the emphasis made on moral responsibility) could enhance pro-environmental behavior among the participants. According to Kaiser et al. (2005), high personal norms of environmental responsibility proved to be a powerful indicator of PEB, such as waste reduction and sustainable consumption. The empirical data always demonstrates that people with high personal environmental responsibility feel moral pressure to participate in PEB (Lu and Wang, 2022; Mackay and Schmitt, 2019; Crandon et al., 2022; Schwarzer and Luszczynska, 2007; Homburg and Stolberg, 2006; De Groot and Steg, 2009; Chen and Lu, 2024; Nolan et al., 2008; Thøgersen, 1996).

*H4*: There is positive relation between personal norms and pro-environmental behavior in China.

The theory of Norm Activation (NAT) states that the personal norms are important factors influencing the development of altruistic and pro-social behaviors. Nevertheless, the conversion of these moral intentions into real actions is usually influenced by the perceived ability of an individual to perform the actions of its wishes (Schwartz, 1977). This is complemented by the Social Cognitive Theory (SCT) which focuses on self-efficacy as an important factor in the enactment of behaviors (Cai et al., 2025c). Empirical studies identified that with strong personal norms towards environmental protection are more likely to engage in sustainable behaviors when they also have high self-efficacy (Cheng et al., 2022; Mar et al., 2023). For example, the intention-action gap is likely to occur in those individuals who perceive that they have a moral obligation to save the environment but are not sure that they can make a significant change (Bamberg and Möser, 2007). On the other hand, a moral motivation of the people to be translated into the actual behavior when they feel that they are able to successfully engage in pro-environmental behaviors like recycling, energy conservation, or waste reduction is higher (Chen and Lu, 2024).

In China, where collective well-being and moral responsibility are major values, self-efficacy may serve as a psychological process that enables people to change the moral consciousness into action in the environmental context. This may be in line with the idea of reciprocal determinism of SCT in which the cognitive (self-efficacy), personal (norms), and behavioral factors are all dynamic. Therefore, the people who view themselves as effective agents have a better chance to live up to their moral responsibility to preserve the environment.

*H5*: Self-efficacy mediates the relationship personal norms and PEB.

**Figure 1:**
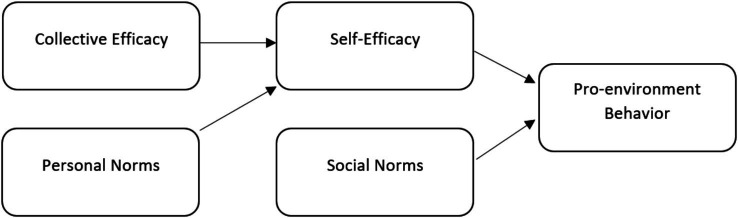
Conceptual framework.

In the context of the Social Cognitive Theory, the collective efficacy and self-efficacy are two different yet interconnected constructs that affect the behavior of human beings. Although self-efficacy is the belief of an individual in oneself to accomplish certain tasks efficiently, collective efficacy is the collective belief of a group of people that they can effectively work together towards accomplishing common goals (Cai et al., 2025c; Bandura, 1986). The two types of efficacy help in the motivation of behavior, although their actions may take a hierarchical approach-collective efficacy reinforces individual self-efficacy, which further increases the chances of engaging in behaviors (Qi et al., 2022). Collective efficacy may establish a positive social environment that contributes to the individual competence and responsibility in the pro-environmental behavior. As people feel that their community, their peers, or their organization can collectively solve environmental problems, they start to internalize such beliefs, which enhances their own feeling of efficacy (Gifford and Nilsson, 2014; Fatima and Azhar, 2021). This self-efficacy increases a motivational intermediary between group faith and personal environmental action. The use of this mechanism is empirically supported, with research indicating that shared perceptions of environmental responsibility positively affect individual pro-environmental behavior in terms of increased personal agency (Cai et al., 2024; Corral-Verdugo et al., 2016). With social cohesion and harmony in the group as central cultural values in China, this mediating relationship is even stronger. Individual confidence is usually preceded by the perception of group ability where individuals are likely to assess their self-efficacy based on the perceived social group competence (Crandon et al., 2022; Ojala, 2018). As a result, those who are confident in their own team to produce environmental outcomes tend to experience individual empowerment to be sustainable and, therefore, connect collective efficacy and PEB to self-efficacy (see Figure 1).

*H6*: Self-efficacy mediates the correlation between collective efficacy and PEB.

The suggested model focuses on the co-existing influence of efficacy beliefs, as well as normative influence, which describes internal motivation and external influence of social pressure processes. Although the current research on pro-environmental behavior has been increasing, not many studies have incorporated both the efficacy beliefs and normative factors in one set of study, especially in the setting of the emerging economies such as China. The paper filled this gaps by empirically confirming an integrated model, which added new information to the overall psychological and social mechanism that leads to sustainable behaviors in non-Western communities.

## 4 Methods

### 4.1 Participants

The data was collected during August 1, 2023—October 30, 2023. The participation in the survey was voluntary and the participants were informed prior to the start of the survey by providing them with a written informed consent. The selection of the participants was based on various online media, such as social media (WeChat, Weibo), emails, and online forums, which enabled the researcher to select both urban and rural members. Despite the fact that the data were mostly gathered using digital channels, including WeChat and Weibo, a combination of various outreach strategies was designed in order to reduce the possible sampling bias in favor of younger or urbanized samples. In order to achieve the demographic and geographic variety, the researchers utilized the email invitation, online community forums, and university networks, as well as the targeted recruitment in urban and rural provinces. These avenues assisted in ensuring that participants were represented in different age groups, income bracket and educational backgrounds. The heterogeneity of the sample was strived to be made by targeting the different provinces and demographics. The participants were given the objectives and the details of the study and in case they were willing to participate, they clicked on I agree. The survey questionnaire was made available to the participants after they had clicked. The questionnaire was made both in Chinese and English and the participants were free to choose the language they wanted to understand. The respondents could complete the questionnaire through their computers or mobile phones. In addition, the participants could quit the survey any time they wished. The target population is also the people living in China and aged above 18 years. The participants of both urban and rural areas were included in the study in order to make the findings to be more representative and applicable to different contexts in China. Out of the sample of 476 final respondents, about 65% of the sample was urban (e.g., major cities Beijing, Shanghai, and Guangzhou), whereas 35% was rural (e.g., villages and small towns, in Hunan, Sichuan, and Inner Mongolia provinces). This balance in the demographics enables the coverage of a wider scope of environmental perceptions and behaviors taking into consideration the fact that a socio-economic, educational, and infrastructural disparity between urban and rural environments can shape pro-environmental behavior. To gather the data, the convenience method of sampling is used. The sampling method was selected because of its convenience and flexibility as it is feasible and available to a wide population in different parts of the country using limited resources and time. This is a common way of getting access to participants who are fast and easy. A survey of 600 questionnaires was completed and the final 476 questionnaires are chosen. The partial and unclear surveys were not assumed to be further empirical analyzed. The sample population was 58% males and 42% females. The age of the participants was between 18 and 60 years with the mean age of approximately 31 years. The percentage of the unmarried participants was 65 and the percentage of the married ones was 35. The sample has a bachelor degree in 44% and master degree in 56%.

### 4.2 Measures

All measurement tools were specially adapted to the Chinese setting in order to have cross-cultural validity with all translation and back-translation procedures. The initial versions of the scales in English were translated into Mandarin Chinese by two bilingual experts in psychology and environmental studies, who independently translated the scales into English to test conceptual equivalence. There were slight linguistic and contextual modifications in order to make the cultural meaning relevant and keep the theoretical meaning of the initial items.

The responses were measured using a five point Likert scale. Self-efficacy and collective efficacy were measured using the scale of perceived climate self-efficacy created by Doran et al. (2015), and the questions in the personal and social norms were adapted from Doran and Larsen (2016). A pilot test of 35 Chinese participants was performed to ensure the suitability of these scales to the Chinese culture before the actual collection of data. The reliability of the pilot results was found to be satisfactory (Cronbach 0.80 or above in all constructs) and indicated that the items were well understood and the context was suitable. Besides, all scales have been validated in literature carried out in Asian setting such as China, making it psychometrically sound in cross-cultural research. All these measures increased the validity and reliability of the adapted measures in the Chinese context. The research examined pro-environmental behavior based on 14 items modified by Ojala (2018). Both individual and collective efficacy was measured using the “perceived climate self-efficacy scale” developed by Doran et al. (2015). This study assessed the individual and social norms regarding climate change using a set of five items for each dimension developed by Doran and Larsen (2016).

Participants’ experience with climate change was assessed using a single item created by keeping in view the work of van der Linden (2015). Climatic Changes Experiences (CCE) was included as a supplementary construct to explore whether individuals’ direct or perceived exposure to climate change correlates with PEB. It was guided by previous findings from van der Linden (2015), who demonstrated that personal experiences with climate-related events (e.g., extreme heat, flooding, droughts) can influence climate change risk perception and increase behavioral engagement. Including this variable allowed us to assess whether experiential knowledge of climate change contributes to PEB in the Chinese context.

## 5 Estimated results

Structural Equation Modeling is used to analyze the data. The main findings are reported in Table 1 while reporting of factor loading of each construct is avoided due to potential length of the study. The value of factor loadings of all constructs are greater than 0.70 which show the reliability of all constructs.

**Table 1:** Reliability analysis.

Item	Factor loading	CR	CA	AVE	KMO	Barlett’s test		
						𝝌^2^	df	Sig
Pro-environmental behavior (PEB)	0.84	0.85	0.92	0.82	0.80	532	4	0.00
Self-efficacy (SLE)	0.86	0.78	0.88	0.81	0.78	486	0.00	0.02
Collective efficacy (CLE)	0.87	0.86	0.93	0.84	0.77	763	5	0.01
Personal norms (PRN)	0.76	0.83	0.84	0.85	0.79	658	4	0.02
Social norms (SLN)	0.83	0.85	0.88	0.83	0.80	567	5	0.01

CR, construct reliability; CA, Cronbach’s alpha; KMO, Kaiser–Meyer–Olkin; AVE, average variance extracted.

The Cronbach’s alpha (CA) values for all constructs exceed the threshold of 0.70, indicating strong internal consistency. Convergent validity is assessed using composite reliability (CR), Bartlett’s test of sphericity, and the Kaiser–Meyer–Olkin (KMO) measure of sampling adequacy. The results demonstrate that CR values surpass the recommended benchmark of 0.80, KMO values are above the acceptable limit of 0.70, and Bartlett’s test is statistically significant at *p* < 0.001, confirming the suitability of the data for factor analysis. Discriminant validity is determined through Pearson’s correlation coefficients and square root of the Average Variance Extracted. The results are presented in Table 2.

**Table 2:** Discriminant validity.

Variable	Pro-environmental behavior	Self-efficacy	Collective efficacy	Personal Norms	Social Norms
Pro-environmental behavior	0.93				
Self-efficacy	0.51	0.80			
Collective efficacy	0.59	0.66	0.79		
Personal norms	0.60	0.51	0.62	0.83	
Social norms	0.66	0.53	0.56	0.50	0.81

All values are significant at 1% while diagonal value is square root of AVE.

The correlation coefficients are lower than the square roots of the corresponding AVE values, indicating that each construct shares more variance with its own indicators than with other constructs, thereby confirming discriminant validity (Doran et al., 2015).

### 5.1 Structural Equation Modeling (SEM)

SEM is used to determine the effects of various factors affecting the PEB. Structural Equation Modeling is a statistical method used to examine the relationship among observed indicators and underlying latent constructs. It enables researchers to evaluate complex theoretical frameworks involving multiple variables and interconnected pathways (Hair et al., 2021). The model is applied using the data, as a measure of how well the observed data emulate the model. SEM estimates the parameters of the model and make overall judgment about how well the model fits the data. The findings are presented in Table 3.

**Table 3:** Structural parameters.

Hypotheses	Path	*t*-value	Result
CLE → PEB	0.542*	5.853	Supported
SLE → PEB	0.489*	6.264	Supported
SLN → PEB	0.618*	7.891	Supported
PRN → PEB	0.587*	6.872	Supported
PRN → SLE → PEB	0.168**	2.742	Supported
CLE → SLE → PEB	0.192**	3.021	Supported
Confirmatory factor analysis			
x^2^/df = 2.857*	RMR = 0.057	NFI = 0.913	IFI = 0.904
CFI = 0.905	RFI = 0.916	GFI = 0.892	RMSEA = 0.075

*, **Shows significance at 1 and 5%, respectively. PEB, pro-environmental behavior; SLE, self-efficacy; CLE, collective efficacy; PRN, personal norms; SLN, social norms.

As suggested by Hair et al. (2021), the structural model results are interpreted in terms of both statistical significance and the strength of relationships. All hypothesized paths were statistically significant at 1%, indicating robust relationship among constructs. The standardized path coefficients (*β* values) demonstrate that social norms (*β* = 0.618) had the strongest influence on pro-environmental behavior, followed by personal norms (*β* = 0.587), collective efficacy (*β* = 0.542), and self-efficacy (*β* = 0.489). Self-efficacy significantly mediated the relationship between personal norms and pro-environmental behavior (*β* = 0.168, *t* = 2.742, *p* < 0.05), as well as between collective efficacy and pro-environmental behavior (*β* = 0.192, *t* = 3.021, *p* < 0.05). These findings indicate that individuals with stronger personal moral obligations toward the environment are more likely to engage in sustainable actions when they also feel confident in their ability to contribute effectively to environmental outcomes. Similarly, collective efficacy enhances pro-environmental behavior not only through shared group beliefs but also by strengthening individual confidence in performing environmentally responsible acts. This partial mediation suggests that self-efficacy acts as an internal psychological mechanism translating moral and collective motivations into concrete environmental actions. The results are consistent with Social Cognitive Theory, which emphasizes self-efficacy as a central determinant of behavioral enactment, and with the Norm Activation Theory, which highlights the role of moral awareness in activating behavioral intentions. These values indicate moderate to strong effects according to the thresholds proposed by Hair et al. (2021), where standardized coefficients above 0.30 are considered meaningful in behavioral research. In addition, model fit indices met the recommended criteria (CFI > 0.90, RMSEA < 0.08), supporting the model’s adequacy. The *R*^2^ value for pro-environmental behavior was 0.68, suggesting that 68% of the variance in PEB is explained by the four predictors—demonstrating substantial explanatory power (Doran et al., 2015). This provides strong empirical support for the proposed theoretical model and emphasizes the utility of integrating social cognitive and norm activation theories.

### 5.2 Correlation analysis

To explore the connections among experiences with climate change, individual norms, social norms, self and collective-efficacy, and PEB, correlation analysis is conducted. The results are reported in Table 4.

**Table 4:** Correlation analyses.

Variable	Climatic changes experiences
Pro-environmental behavior	0.33**
Individual norms	0.61
Personal norms	0.66**
Individual self-efficacy	0.52**
Collective efficacy	0.51**

**Show significance at 5%.

The correlation analysis has demonstrated that all major constructs such as self-efficacy, collective efficacy, personal norms, and social norms were positively correlated with PEB, which can confirm the hypothesized relationships. It is worth noting that the highest levels of correlation were found between social norms and PEB, and between personal norms and PEB, meaning that the normative factors are extremely important in forming the sustainable behaviors of the Chinese. There were also some weak relationships. The correlation between self-efficacy and PEB was positive by the weaker association compared to social and personal norms. This may be a sign of the collectivistic Chinese culture whereby social approval and influence of the community are strong movers of behavior change. This is consistent with facts that within collectivist societies, human beings are more responsive to social pressures and align their actions to conform to social expectations to maintain harmony and avoidant social penalties (Doran and Larsen, 2016; Hair et al., 2021). In that kind of environment, behavior is not only prone to be affected by intrinsic agency but to behave as perceived by social norms and be liked by other people (Triandis, 1995; Yang et al., 2021; Hogg and Turner, 1987). The fact is also supported by the earlier research. Cheng et al. (2022) showed perceived social norms were more effective in defining green consumption behavior amongst Chinese millennials compared to individual values or environmental knowledge especially when the social media was used to reinforce normative behavior. On the same note, Wang and Wang (2021) established that peer and family expectations were very effective in improving green commuting behavior even in cases where personal confidence (self-efficacy) was moderate (Homburg and Stolberg, 2006; Hogg and Turner, 1987). Likewise, collective efficacy that was considerably related to PEB, did not demonstrate as strong a relationship as social norms. This could be in support of the fact that although people believe in the ability of the group to act as one, there is still a possibility of a great dependence of an individual in the perceived social norms to determine whether to participate in an environmentally friendly action. As mentioned in the Social Cognitive Theory (Bandura, 2000), collective efficacy increases group motivation and coordination and promotes pro-environmental behavior. Nevertheless, in interdependent cultures, it is common to find people aiming at matching social expectations in a bid to maintain group harmony and in order to gain social approval (Doran and Larsen, 2016; Wang and Wang, 2021). Geiger et al. (2020) noted that descriptive norms are more influential on environmental decision-making than internal efficacy beliefs, in the high social surveillance situation. These results are aligned with the normative action theory (Cialdini et al., 1990; Cialdini and Jacobson, 2021), which states that people tend to be guided by the norms in order to receive social approval and escape rejection. Therefore, collective efficacy leads to group-level motivation, yet the powerful behavioral signal might be provided by the immediate social pressure and the perceived behavioral expectations in one of the reference groups. Altogether, the results point to the fact that normative pressure can have a direct stronger impact on pro-environmental behavior as compared to personal or group efficacy beliefs in Chinese context.

## 6 Discussion

This paper investigated the relationship among self-efficacy, collective efficacy, personal and social norm in China by collecting the data from 476 personals and conducted the empirical analysis through SEM. According to the empirical results, the collective efficacy has a positive influence on the PEB, which supports *H1*. When a belief is held by a group that it can exercise its joint power to make changes, the common belief may extend to motivate the group members to change their behaviors and actions in a direction that will meet the group’s goals, such as pro-environmental behaviors (Harland et al., 1999). It enhances the moral obligations and shared responsibility, which motivate the people towards PEB that are more consistent with the values and norms followed through the group (Qi et al., 2022; Ojala, 2018). This boosts group members’ motivation and commitment towards pro-environmental goals. If individuals, see their peers actively involved they will more likely to contribute with their efforts (Doran et al., 2015). A high-efficacy group’s sense of belonging and peer support might also help to provide encouragement and reinforcement for developing and retaining pro-environmental behaviors. Social cohesion is a prerequisite for many collective actions, including community cleanups, energy-conservation campaigns, and advocacy efforts (Doran and Larsen, 2016). When a group has faith in the power of its collective actions, members would more willingly distribute their responsibilities so that there is a working mechanism to complete an environmental project and even to find more effective solutions. Groups characterized by high collective efficacy will be more prone to sharing information and social learning processes. Members start sharing information about the best practices in general, novel solutions, and techniques to preserve the environment (Hair et al., 2021). Groups high in collective efficacy are better able to organize various resources, including time, money, and materials, needed to engage in pro-environmental activities. This may range from fund-raising to acquisition of grants, or it could entail pooling community resources to finance and support environmental projects (Triandis, 1995). Through collective efficacy, the group is made more competent on organizing events or conducting educational programs and actually accomplishing major environmental projects. Collective efficacy is a very strong support for enhancing pro-environmental behavior through the increase in motivation and social cohesiveness, information sharing, and mobilization of resources. The theoretical framework, such as the social cognitive model, TPB, or VBN model, supports the evidence from community-based programs and environmental activism, so it can be said that the impact of collective efficacy on PEB is positive.

The *H2* is also supported that self-efficacy enhances the pro-environmental behavior. High self-efficacy enhances personal motivation to engage in PEB. Individuals present strong intentions with lower rates of counter intention to perform pro-environmental behavior, and are less sensitive to changing situational constraints. In addition, they exhibit lowered sensitivity to changing levels of resistance and difficulties presented in achieving environmental goals, leading to persistent behavior in the face of setbacks and/or difficulties (Yang et al., 2021; Park et al., 2024). Self-efficacy is related to an improved ability to solve problems. Persons with high self-efficacy propose some effective solution to an environmental problem, and they succeed in doing so. High self-efficacy will allow individuals to change their way of circumstances and find new alternatives concerning how to undertake pro-environmental behaviors (Zhang et al., 2023). Self-efficacy accounts for positive emotional reactions, such as satisfaction, pride, and a sense of accomplishment. These feelings serve as rewards to encourage the repetition of pro-environmental acts. High self-efficacy will significantly minimize stress and anxiety in regard to environmental challenges, making an individual stronger and more psychologically capable of taking constructive action (Kumar and Singh, 2023). People with higher level of self-efficacy usually ripen into exemplary models for imitation, demonstrating effective pro-environmental behaviors and inspiring other people to take environmentally friendly actions. When self-efficacy is high, one is able to enhance social support from peers, family, and neighbors in fostering an environment that is more responsive to pro-environmental measures (Park et al., 2024; Zhang et al., 2023; Kumar and Singh, 2023; Li et al., 2024). Self-efficacy plays a significant role in the assurance of PEB by enhancing motivation, problem-solving skills, emotional well-being, and sources of influence. The role of self-efficacy in actual environmental action has also been already supported by many existing theoretical frameworks, along with empirical data from other studies. Problems with overestimation and features of the context factors are to be solved optimally in an attempt to maximize the beneficial effects obtained by self-efficacy in environmental change. Making actions more empowered, purposeful, and lasting will, therefore, be better through the application of self-efficacy strategies: for example, environmental education, participation by community members, and role modeling. Our results are consistent and in line with the primary assumptions of the Social Cognitive Theory (van der Linden, 2015; Cai et al., 2025a) and its implementation in various environmental researches in different cultural settings (Bandura, 1986). This consistency highlights the importance of how a person believes in their potential to take environmental measures. Likewise, the strong positive role of personal norms on PEB is highly reminiscent of the predictions based on the Norm Activation Theory (Stern, 2000; Cai et al., 2025b) and the results obtained in the other studies (Bamberg and Möser, 2007) which highlight the role of personal moral obligation in the motivation of a sustainable behavior.

The *H3* is substantiated that social norms play a strong and positive role in influencing PEB. This relationship can be viewed in a number of channels. Social norms give the feeling of obligation and responsibility in individuals to perform those behaviors that are deemed socially desirable or acceptable under their community or social group (Lu and Wang, 2022; Mackay and Schmitt, 2019). When others think that people in their societal network are performing eco-friendly behavior, they imitate them in a bid to fit in these patterns and be socially accepted (Crandon et al., 2022). Secondly, social norms affect behavior as they determine the beliefs held by people concerning what is right or effective in solving environmental problems (Gardner and Stern, 2008; Al-Ghazali and Abdulraheem, 2024). When people see that their fellows believe in and care about the preservation of the environment, they adopt the views and integrate them in making their decisions (Doherty and Clayton, 2011). People argue that social norms are great motivators of pro-environmental behavior because they impact on individual’s beliefs, attitudes and behaviors in a way that will be able to enhance environmental conservation and sustainability (Homburg and Stolberg, 2006).

Although our findings validate the already known significance of social norms in PEB (Cialdini and Jacobson, 2021; Homburg and Stolberg, 2006), a significant observation is the high level of collective efficacy observed in this study. The effect of collective efficacy supports the current studies that highlight the significance of group capability beliefs in the environment (Fritsche et al., 2018). However, the comparative strength of collective efficacy that is observed in our model, as well as the high impact of social norms, seems to be especially typical in Chinese setting. Such a trend implies that in collectivist cultures such as China, where the importance of group harmony and interdependence takes a central role (Doran and Larsen, 2016; Sander and Ulrich, 2024), the collective efficacy and the perceived group pressure (social norms) may have a stronger impact on individual PEB. It is reasonable to suggest that the prevalence of collective efficacy and social norms in PEB may be attributed to a number of interconnected characteristics of the modern Chinese socio-cultural and political environment. To begin with, the strong traditions of collectivism of the Chinese culture focus on group objectives, social cohesion, and adherence to common expectations (Lee et al., 2025), which inherently increases the effect of the collective level, such as collective efficacy beliefs and social norms. Second, the high priority of the Chinese government towards national environmental agendas, which is embodied in such frameworks as the Ecological Civilization creates an environment, in which top-down policy efforts usually depend on and strengthen community mobilization and collective responsibility. Furthermore, rapid urbanization in China has increased reliance on community-level management for implementing environmental policies (e.g., household waste sorting programs), making perceptions of collective capability and prevailing community norms especially salient for individual participation.

The *H4* is also supported from empirical analysis that self-efficacy positively affects the pro-environmental behavior because individual with high self-efficacy set goals related to PEB make efforts to achieve. They believe that their actions make a difference, leading to a greater commitment to environmental conservation (De Groot and Steg, 2009; Li et al., 2024). High self-efficacy individuals view obstacles or setbacks as challenges to be overcome rather than insurmountable barriers. They are more adept at finding creative solutions and adapting their behaviors to achieve environmental goals (Chen and Lu, 2024; Al-Ghazali and Abdulraheem, 2024; Sander and Ulrich, 2024). They are often seen as role models by others. Their assurance of performing PEB can motivate and encourage others to follow same, generating a ripple effect in their social circles. Individuals with high self-efficacy are better able to view risks of PEB as more controllable and hence are more inclined to act. They are less likely to be dissuaded by anticipated hindrances or unfavorable results (Nolan et al., 2008; Lee et al., 2025). They are more efficient in regulating their feelings in the face of environmental threats. They are more effective when controlling their emotions when confronted with environmental dangers. They will experience less discouragement and frustration which leads to consistency and persistence of behavior in PEB (Clayton and Karazsia, 2020; Schwarzer and Luszczynska, 2007; Thøgersen, 1996). Having high self-efficacy would make individuals to develop a strong environmental identity where they view themselves as custodians of the environment. This is closely related with their actions and behavior which further reinforces their support in PEB (Fatima and Azhar, 2021; Harland et al., 1999).

The outcomes of the mediation analysis give a more profound understanding of the psychological mechanism through which the normative and collective intentions are transformed into pro-environmental behavior. The findings that self-efficacy mediates personal norms and collective efficacy implies that moral knowledge and collective beliefs would be inadequate to create sustainable behaviors except when individuals also hold their personal ability to act to create a difference. This is in line with the Social Cognitive Theory, which emphasizes that behavior change will gain more ground when people feel that they can perform the activities that will result in the expected changes in the environment (Bandura, 1986; Hofstede, 2001). According to the Norm Activation Theory, moral obligations (personal norms) are activated in the behavioral intentions only with the assistance of the sense of self-competence. Additionally, collective efficacy improves self-efficacy in a collectivist society such as China where group harmony and social responsibility are fully integrated to make people believe that they share confidence and have a moral obligation to conserve the environment (Gardner and Stern, 2008; Wu and Liang, 2024). These dynamics hint that the intervention directed at the development of the pro-environmental behavior must emphasize the reinforcement of individual competence beliefs as part of group-based intervention such as community-based environmental education, public acknowledgment of the local environmental activities, and the education campaigns that would be characterized by the combination of moral appeals.

Even though the Climate Change Experience (CCE) did not feature in the theoretical model of the study, it was included in the correlation analysis for further contextual information. The moderate interrelation between CCE and pro-environmental behavior implies that the personal experience of climate-related occurrences may increase environmental awareness, but does not necessarily contribute to behavioral change when there is no significant cognitive and normative support. This observation is consistent with other studies (van der Linden, 2015) demonstrating that direct climate experiences raise perceived risk and necessitate the psychological processes like efficacy beliefs and moral norms as motivational variables. Experience awareness may not be sufficient to initiate change in the Chinese collectivist cultural setting unless it is supported by common norms and collective efficacy that would turn awareness into coordinated pro-environmental action. Hence, CCE is not the cause, but a supplementary-based factor, indicating the significance of experiential, cognitive, and social aspects acting in tandem to shape sustainable behaviors.

### 6.1 Theoretical contributions

The study adds to the literature by providing an integrated view on the effects of efficacy belief and normative influences in shaping environmental behavior, which was not well explored in the past, particularly in non-Western societies like China. It shows the correlations between individual and social motivational variables and empowers the efficacy and norm-based theories to be valid in the explanation of PEB simultaneously. Additionally, the research contributes to the existing literature on cross cultural environmental psychology as it puts findings in the fast changing socio-economic environment of China into perspective.

The social cognitive theory is also supported in this study as the influence of social constructs like collective efficacy as well as social norms on PEB is shown. It underlines the importance of the beliefs based on group efficacy and social influence in encouraging people to perform environmental friendly actions. It is worth noting that the findings show how social norms are perceived to have power to drive PEB. The study enhances the understanding on normative effect on behavior in revealing that the perceived social group value for environmental preservation results in experience greater probability to perform a PEB. The study highlights how collective efficacy presumes mass action for environmental change. This is an indication that groups which have faith in their capacity to achieve some shared objectives make efforts to meet environmental challenge.

### 6.2 Practical implications

**Figure 2:**
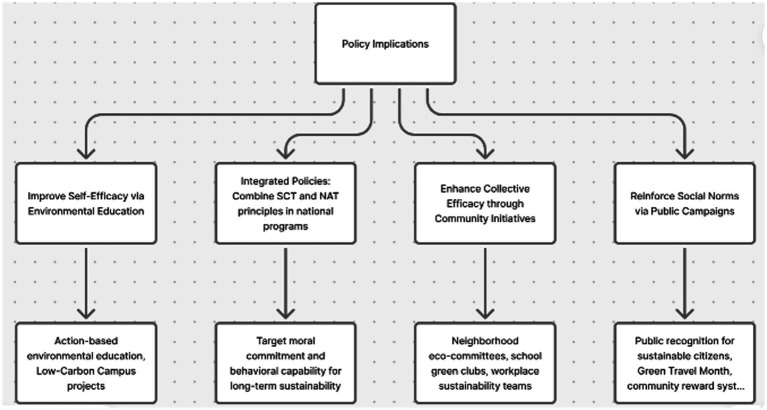
Practical implications.

These findings indicate that additional resources are to be redirected to support the improvement of collective efficacy beliefs and positive social norms ascribed to the environmental protection in China. The campaigns may focus on the power of the collective action in terms of sharing values by community to promote PEB. These campaigns can be initiated using the social media and educational programs on the power and significance of collective efficacy and social norms in terms of the fight against the environmental challenges. Furthermore, school and university education programs on environmental education might not only be based on knowledge sharing but also on the development of self-efficacy based on action-oriented, practically based projects. An example is the introduction of sustainability audits and adoption of environmentally friendly behavior by students at the Guangdong province with the development of the Low-Carbon Campus projects which have enabled students to be self-efficacious in their environmental domains. A conducive situation of PEB can be established through promoting collective action, e.g., by rewarding sustainable behavior. As an example, local environmental programs, like recycling contests in the neighborhoods like Shanghai, which has been well executed, may have some effect in instilling a sense of communalism and communal success. Policies that foster the creation of shared responsibility and emphasis on extensive sustainable practices in communities can greatly increase the scope of the environmental response. Behavior change messages should also be incorporated in the larger climate policies by policymakers, and this should focus on the idea that individual and collective behavior, however small, has a significant role in protecting the environment, which proves to be an effective strategy in the Singaporean campaigns of the Sustainable Singapore Blueprint. An implication trail in the Figure 2 is shown below.

Given the mediating effect of self-efficacy between the personal norms and collective efficacy, the policy should be designed so as to balance between the empowerment based approaches and meeting the sustainability objectives. Second, as collective efficacy enhances encourages collective action, group based forces like neighborhood eco-committees, school-based green clubs or workplace sustainability teams can be a potent means of developing collective responsibility and observable social norms of environmental protection. Lastly, the social cognition theory and norm activation theory should be incorporated in environmental education programs to instill moral commitment and behavioral competency in the younger generations. Through the combination of the normative motivations and self-belief systems, the policymakers can develop a more psychologically-grounded, as well as culturally-resonant mechanism of attaining a long-term sustainability of the environmental sphere.

### 6.3 Limitations and future research

The sample of the research is not representative of the overall population of China since the research method used to gather data is the convenience sampling method. This restricts the generalization of results. Stratified random sampling can be used in future research to make the sample more depictive of the population in different regions of China. Besides, the analysis of self-reporting data, which is prone to social desirability bias, and does not necessarily indicate the true behavior of participants or beliefs. Moreover, the results might be limited to the Chinese cultural and socio-economic environment and might not apply to other nations and areas. The differences in context of the drivers of environmental behavior would also be examined by conducting comparative studies among urban and rural environments. Further work that includes behavioral observation techniques or objective indicators of environmental behavior (e.g., a record of household energy consumption or waste management) could enhance the accuracy of data. In addition, even though the research is conducted in China, which is a collectivist culture with its own cultural and policy dynamics, future studies may consider adopting other collectivist cultures in order to evaluate the cross-cultural applicability of the integrated Social Cognitive Theory and Norm Activation Theory model: like Japan, South Korea, or Indonesia. The similarity/dissimilarity of the roles of collective efficacy and social norms across the various cultural orientations would be established by making comparative studies of the individualistic and collectivist cultures (e.g., China vs. the United States). In addition, longitudinal research designs would give great information on how pro-environmental behavior changes over time due to the variations in policy interventions, environmental awareness campaign, or changes in collective efficacy and social norms. Mechanisms of behavioral patterns can be observed over time and may lead to determination of the persistence and sustainability of environmental behaviors, and the turning points in the patterns of the engagement of the individuals with the environment. Lastly, future research could examine moderating or mediation effects of cultural and contextual factors, such as collectivism, environmental concern, or governmental trust, between efficacy beliefs, social norms, and pro-environmental behavior. The coverage of these areas would enhance the theoretical concept of pro-environmental behavior and reinforce the practical implications with regard to promoting the sustainability of various socio-political settings.

## 7 Conclusion

The purpose of this study was to examine the psychological and social determinants of pro-environmental behavior in China, such as the self-efficacy, collective efficacy, personal norms, and social norms. Based on the principles of the Social Cognitive Theory and Norm Activation Theory, the study hypothesized and tested an integrated model to learn how beliefs and social expectations of individuals interact to affect environmentally responsible behaviors. The research with a sample size of 476 Chinese respondents established that all four variables have a significant positive relationship with PEB through structural equation modeling. Moreover, self-efficacy has mediating role in these relationships. Such findings provide a better insight into the aspects that influence sustainable behavior, especially in the socio-cultural environment of China.

Among the examined variables, social norms had the most substantial impact on PEB. This highlights the tremendous impact of perceived expectations in shaping individual action. In a collectivistic society such as China, where social conformity, mutual obligations, and communal well-being are deeply embedded in cultural norms, individuals are highly responsive to the behaviors and attitudes of others. The perception that others are engaged in environmental friendly practices can act as a powerful behavioral cue, reinforcing the social acceptability and desirability of such actions. Personal norms also emerged as a strong predictor of PEB. These moral obligations reflect personal values and ethical commitments to environmental protection. When individuals believe that taking care of the environment is a matter of personal responsibility, they engage in consistent pro-environmental actions, even in the absence of external incentives or pressures. This finding suggests that cultivating moral awareness and a sense of environmental duty at the individual level is critical for promoting sustainable behavior.

Collective efficacy also showed a significant positive relationship with PEB. This suggests that when individuals perceive their communities or social groups as capable of effectively addressing environmental challenges, they are motivated to participate in collective actions such as recycling programs, energy conservation initiatives, or public clean-up efforts. In this sense, collective efficacy acts as a bridge between individual intent and communal action, reinforcing the idea that environmental sustainability is a shared goal that requires joint efforts. Self-efficacy also had a significant and positive association with PEB. This elaborated the importance of individual agency and the belief in one’s ability to perform specific tasks related to environmental sustainability. Individuals with high self-efficacy are more likely to initiate and maintain environmental friendly behaviors, such as reducing water usage, using public transportation, or minimizing plastic consumption. Although its effect was weaker compared to social or collective influences, self-efficacy remains a critical component of behavioral intention and persistence, especially in contexts where individual action is required.

These findings highlight the critical role of social and normative influences in shaping sustainable behavior, suggesting that both personal beliefs in one’s capabilities and the perceived expectations of others are essential in motivating individuals toward environmental responsibility. The results provide empirical support for an integrated theoretical framework and reinforce the importance of designing policies and interventions that strengthen both efficacy beliefs and normative commitments.

## Data Availability

The original contributions presented in the study are included in the article/supplementary material, further inquiries can be directed to the corresponding author.
